# A Study of Mechanisms of Nanobubble-Enhanced Flotation of Graphite

**DOI:** 10.3390/nano12193361

**Published:** 2022-09-27

**Authors:** Fangyuan Ma, Dongping Tao

**Affiliations:** 1 School of Mining Engineering, University of Science & Technology Liaoning, Anshan 114051, China; 2 School of Resources and Environmental Engineering, Shandong University of Technology, Zibo 255049, China

**Keywords:** adsorption, enhancement mechanisms, flotation kinetics, graphite, nanobubbles

## Abstract

This study was conducted to investigate the mechanisms of enhanced microfine flake graphite (MFG) flotation by nanobubbles generated based on the principle of hydrodynamic cavitation. The effects of nanobubbles on graphite surface properties were characterized in terms of the flotation kinetics, collector adsorption behavior, Zeta potential, IR spectra, contact angle, etc. The results show that the surface nanobubbles increased the hydrophobic attraction and reduced the electrostatic repulsion between the graphite particles and collector molecules, significantly improving the flotation selectivity and the kinetic flotation rate and promoting the agglomeration of MFG.

## 1. Introduction

Natural graphite is a crystalline carbonaceous compound with a layered structure. Although it is a non-metallic material, it has both metallic and non-metallic characteristics. Its metallic characteristics include a high rate of thermal conductivity and electrical conductivity [1]. Graphite is characterized by a density of 2.25 g/cm^3^, a melting point of 3652 °C, and a boiling point of 4827 °C. Graphite possesses very stable chemical properties with high resistance to corrosion, acid, alkali, and other agents. Natural graphite can be divided into three major types, namely flake graphite, lump graphite, and microcrystalline graphite, and each has some unique characteristics [2]. Graphite is widely used in batteries [3,4], electrodes [5], conductive coatings [6], lubrication [7], refractories [8], neutron moderators [9], seal materials [10], etc. In particular, the discovery of graphene has promoted new applications of graphite in areas such as sensors [11] and electrodes [12].

Compared with other types of graphite, flake graphite has better floatability, plasticity, and lubricity, but its raw ore grade is usually low. Flake graphite can readily produce a high-grade, high-value graphite concentrate of 95% or higher purity through repeated grinding and multiple stages of separation in a variety of industrial applications, which has been well-demonstrated in China [13]. Based on its grain size, the flake graphite can be further divided into fine flake graphite (flake sizes smaller than 147 μm) and large flake graphite (flake sizes greater than 147 μm) [14]. Fine and large flake graphite can co-exist in the ore body, and in fact, the raw ore of fine flake graphite usually contains approximately 20% large flake graphite. Fine flake graphite will be the main form of graphite resource in the future due to its large reserve. There exists a rich deposit of fine flake graphite in the Luobei region of Heilongjiang Province, China, which contains up to 70% −74 μm graphite in the raw ore [15] and is often called microfine flake graphite (MFG).

Due to the tiny grain size of fine flake graphite, it is difficult to collect all the fine graphite particles in the flotation process, resulting in poor recovery. Therefore, many researchers have attempted to improve the flotation process and chemical reagents to more effectively recover these fine particles of graphite. Bu et al. (2018) showed that the use of a static microbubble cyclone flotation column could increase the recovery of fine flake graphite by 9.59 percentage points at the same ash content of concentrate, compared with a traditional mechanical flotation machine [16]. Bu et al. (2018) also showed that the ash removal efficiency of the flotation column was 3.82-fold higher than that observed with the mechanical flotation cell [17]. Kang and Li (2019) showed that ultrasonic treatment can shorten the graphite cleaning flotation process [18] by efficiently removing silicate impurities as well as other metal impurities. Shi et al. (2015) concluded that better emulsion stability results in smaller diesel droplets in its emulsion, thus improving the recovery of graphite [19].

Ma et al. (2021) reported that nanobubble flotation technology improved the flotation recovery of fine flake graphite by up to 14.73 percentage points and significantly reduced the required number of flotation stages to produce the specified concentrate grade [15]. Similar conclusions have been reached in a fairly large number of studies performed with other coal and minerals, as has been documented in a recent review article by Tao [20]. The separation mechanisms of ultrafine minerals by nanobubbles are mainly related to the exceedingly large contact angle of surface nanobubbles [21,22,23], the hydrophobic agglomeration of particles [24,25], the selective nucleation of nanobubbles on the hydrophobic interface [26,27], the stability of nanobubbles [28,29,30], etc. However, there are few studies focused on the effects of nanobubbles on the particles’ surface properties such as their surface potential, collector adsorption capacity, and adsorption kinetics.

In our previous studies, it has been concluded that the use of nanobubbles can significantly enhance the flotation of microfine flake graphite [15]. Since the mineralization efficiency during flotation is dependent on the adsorption of the collector on the mineral surface, it is of great significance to study the adsorption characteristics of the collector on mineral surfaces in the presence of nanobubbles. However, the adsorption performance of the collector on graphite surfaces has not been thoroughly studied by researchers. This study was intended to reveal the enhancement mechanisms of nanobubbles on MFG from the perspective of the collector adsorption behaviors on graphite and bubble surfaces and their consequent effects on surface characteristics.

## 2. Experimental

### 2.1. Sample

The representative samples of microfine flake graphite ore (MFGO) were collected from the pulp stream of a concentrator in Luobei County, Heilongjiang Province, China. The sample was subsequently filtered, mixed, and thoroughly homogenized before it was fractionated into small lots and stored in sealed bags for later usage. A pure microfine flake graphite (PMFG) sample with a 99.5% fixed carbon content was purchased from Diyuan Graphite Co., Ltd. (Hegang, China).

### 2.2. Sample Characterization

A sample of 2 g MFGO blended with paraffin solvent was employed for the quantitative mineralogical analysis with the Automatic Mineral Identification and Characterization System (AMICS ZEISS Sigma 500) purchased from Bruker, Germany. An X-ray diffraction (XRD) diffractometer (Bruker D8 XRD) was used for the characterization of PMFG under conditions of 40 kV, 44 mA, and 2°/min scan rate. A laser particle size analyzer (BT-9300S) manufactured by Bettersize Instrument Ltd., Dandong, China, was adopted for the particle size distribution characterization of MFGO and PMFG. The particle size distribution curves of MFGO were obtained under the shading rates of 12.31% and 14.56%, respectively.

**Figure 1:**
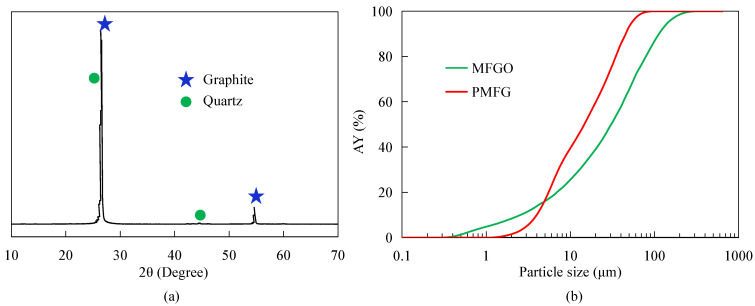
The XRD analysis results of PMFG (**a**) and particle size distribution results of MFGO and PMFG (**b**).

Table 1 shows the AMICS mineralogical identification results of MFGO. It can be seen that the sample had 84.06% graphite, and muscovite, quartz, and pyrite were the main gangue minerals. In addition, the sample also contained a small amount of garnet, calcite, and potassium feldspar. The XRD analysis results shown in Figure 1a indicate that PMFG contained some trace amounts of quartz in addition to graphite. The particle size distribution results shown in Figure 1b demonstrate that the −74 μm content values in MFGO and PMFG were 78.5% and 99.50%, respectively, and d_50_ values in MFGO and PMFG were about 32 μm and 15 μm, respectively.

### 2.3. Flotation System

**Figure 2:**
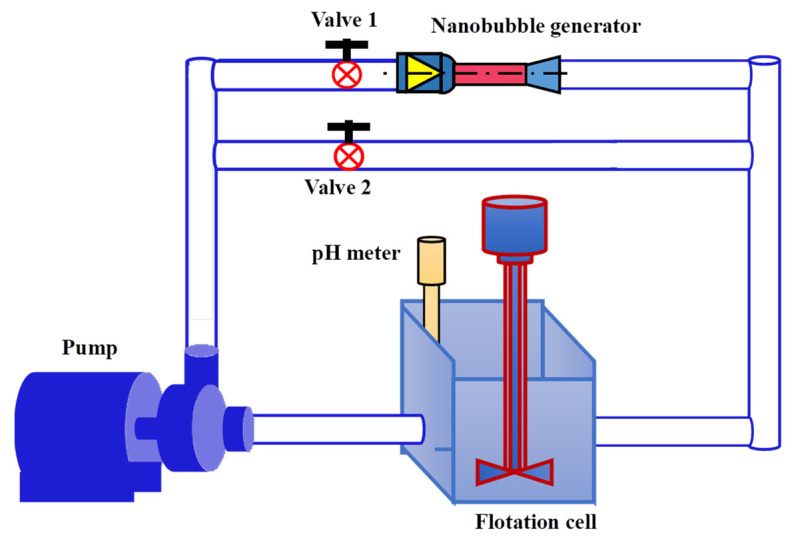
The nanobubble flotation system used in this study.

The nanobubble flotation system used in this study is shown in Figure 2. It consisted of a circulating pump, a nanobubble generator, and a mechanic flotation machine. The circulating pump was produced by Zhejiang Common People Pump Co., Ltd. (Wenling, China), and the flotation machine (XFD 1.0 L) was acquired from Jilin Exploration Machinery Factory. The nanobubble generator with a throat of 3 mm in diameter was used to generate the nanobubbles with the details described in a previous study [31]. When valve 1 was open, and valve 2 was closed, the circulating pump would transport the pulp through the nanobubble generator to produce the nanobubble flotation pulp (NFP) and nanobubble flotation concentrate (NFC). In contrast, when valve 1 was closed, and valve 2 was open, the conventional flotation pulp (CFP) and conventional flotation concentrate (CFC) would be generated with the circulating pulp passing through the steel tube. This flotation system was also employed to generate the samples used to study the adsorption behavior of the collector on graphite surfaces with and without nanobubbles. It is worth noting that the surface nanobubbles and bulk nanobubbles can be generated simultaneously on the surface of graphite particles and in aqueous solutions, respectively, when an aqueous solution with added frother is circulated through the nanobubble generator; these processes have been described by Ma et al. (2019) [32] and Ma et al. (2022) [33].

### 2.4. Bubble Size Measurement

The bubble size was measured with a laser particle size and Zeta potential analyzer (Malvern Zetasizer Nano-ZS90) manufactured by Malvern Panalytical, England. An aqueous bulk nanobubble solution was obtained from the system shown in Figure 2 under the following conditions: valve 1 open, valve 2 closed, pH 6, liquid circulation rate 18.34 L/min, and circulation time 2 min. A 3 mL sample of the aqueous solution was collected from the flotation cell with a disposable plastic syringe and subsequently transferred to the Malvern Zetasizer Nano-ZS90 analyzer to obtain the size distribution characteristics of nanobubbles. It should be noted that nanobubbles can survive for a long time, e.g., hours or days depending on solution conditions, and therefore, it is feasible to use the above particle size analyzer to determine the size distribution of the bubbles in a testing period of a few minutes [31].

### 2.5. Kinetic Flotation Tests

The flotation reagents used in this study were acquired from Heilongjiang Luobei Diyuan Graphite Company in China. All the flotation tests were carried out under the following conditions: frother: fusel 300 g/t, collector: diesel 400 g/t, pH 10 (adjusted with lime), quartz and silicate depressant: sodium hexametaphosphate 1000 g/t, pulp solids concentration 10% by weight, pulp-stirring rate 1800 r/min, pulp circulation rate 17.38 L/min, and flotation time 3 min. The reagents were added into the pulp in the sequence of lime, sodium hexametaphosphate, diesel, and fusel, with an interval of 2 min each. It should be mentioned that the pulp had been well-circulated and mixed for 3 min with valve 1 closed and valve 2 open before reagent conditioning was initiated. It also needs to be mentioned that the lime was directly added to the pulp, and the pH of the pulp was measured in real time using a pH meter until it reached pH 10 before the addition of lime was stopped. Lime has two functions: one is to ensure that the flotation is carried out at the optimum pH, and the other is to depress pyrite in the slurry system. The following reactions can occur after quicklime is dissolved in the slurry [34]:

(1) $$\mathrm{CaO}+H_{2}O\to \mathrm{Ca}{\left(\mathrm{OH}\right)}_{2};$$

(2) $$\mathrm{Ca}{\left(\mathrm{OH}\right)}_{2}\Leftrightarrow \mathrm{Ca}{\left(\mathrm{OH}\right)}^{+}+{\mathrm{OH}}^{-};$$

(3) $$\mathrm{Ca}{\left(\mathrm{OH}\right)}^{+}\Leftrightarrow {\mathrm{Ca}}^{2+}+{\mathrm{OH}}^{-};$$

There are two theories about the depression of pyrite by lime. One is that lime depresses pyrite as a result of the formation of hydrophilic Fe(OH)_2_ and Fe(OH)_3_ films on the mineral surface. The other theory postulates that the depression of pyrite by lime is due to the formation of the hydrate films of CaSO_4_, CaCO_3,_ and CaO on the pyrite surface.

### 2.6. Adsorption Kinetics of Diesel on Graphite Surface

The adsorption characteristics of diesel on the graphite surface in the presence and absence of nanobubbles were investigated with the system shown in Figure 2 under the following conditions: pulp-stirring rate 1500 r/min, pH10, fusel dosage 10 mg/L pulp, pulp solid concentration 30 g/L, and pulp circulation rate 12 L/min. Distilled water was used for pulp preparation. The PMFG pulp samples of 10 mL in volume with different diesel concentrations with and without nanobubbles were collected and filtered. The filtrate was diluted 10 times after 10 min ultrasonic treatment to ensure the diesel content was well-dispersed. Finally, the residual content of organic carbon in the filtrate was determined with a TOC analyzer (TOC-L TNM-L CSN, Japan). Since the main element in diesel is organic carbon, the adsorbance of organic carbon in diesel was used to evaluate the adsorption density of diesel on the graphite surface, as shown in Equation (4):

(4) $$q=\frac{\left[C_{1}-\left(C_{2}-C_{3}\right)\right]V}{m}$$

where ***q*** is the diesel adsorption capacity by graphite (mg/g); *C*_1_ is the initial concentration of organic carbon of diesel (mg/L); *C*_2_ is the concentration of organic carbon in the filtrate (mg/L); *C*_3_ is the concentration of organic carbon in fusel (mg/L). *V* is the volume of distilled water in the whole system (L); *m* is the mass of graphite in the pulp (g). Each measurement was repeated 5 times, and the average value was used as the organic carbon concentration for data analysis. The organic carbon concentrations in fusel and diesel were measured prior to each adsorption test to minimize experimental errors.

### 2.7. Zeta Potential Measurement

In order to characterize the effect of electrostatic interactions on the nanobubble flotation of MFG, the surface potentials of the untreated PMFG particles, bulk nanobubbles, PMFG particles with surface nanobubbles, and with the diesel content were measured by the use of a Zeta potential analyzer (Malvern Zetasizer Nano-ZS90) at pH 10. The surface potential of the untreated PMFG particles was determined with a small sample ground to below −45 μm and placed in distilled water at 3% solid concentration by weight. The bulk nanobubbles were generated using the system shown in Figure 2 at a fusel concentration of 30 mg/L in the solution with valve 1 open and valve 2 closed, and a 5 mL nanobubble aqueous solution sample was collected using a disposable plastic syringe after the system was operated for 2 min. The nanobubble- and diesel-treated PMFG samples were prepared with the same flotation system under the same operating conditions after the addition of a well-dispersed emulsion of 150 μL diesel in 150 mL 1 mmol/L NaCl solution [35] prepared with an intense agitation and ultrasonic treatment for 30 min [36].

### 2.8. Contact Angle and FTIR Measurement

The PMFG contact angle and FTIR measurements were performed with the froth products obtained using the flotation system shown in Figure 2 in the presence and absence of nanobubbles at pH10 with 1000 g/t sodium hexametaphosphate, 10 mg/L diesel, 20 mg/L fusel, 5% solid concentration by weight, and 3 min flotation time. The froth products were washed, dried at 40 °C for 10 h, and pressed into thin plates for contact angle and FTIR measurements with a contact angle analyzer (JC2000C1) produced by Shanghai Zhongchen Digital Technology Instrument Co., Ltd. (Shanghai, China), and a Fourier infrared spectrometer (VERTEX 80 V Bruker) operating at a vacuum pressure of 400 Pa, respectively.

## 3. Results and Discussion

### 3.1. Nanobubble Size Distribution

**Figure 3:**
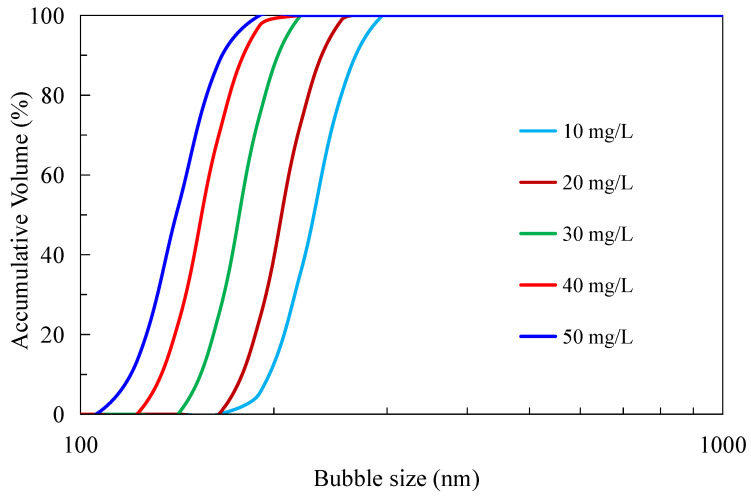
Nanobubble size distribution at different frother concentrations at pH 6.

Figure 3 shows the size distribution of the bulk nanobubbles in the aqueous solutions at different frother concentrations ranging from 10 to 50 mg/L. It can be seen that the average size of nanobubbles decreased from 230 nm to 140 nm with the increases in the frother concentration from 10 mg/L to 50 mg/L, which is consistent with the results reported by Oliveira et al. (2018) and Ma et al. (2019) [31,37]. Finch et al. (2008) showed that increasing the frother concentration reduced the surface tension, which was responsible for reduced bubble size [38]. Zhang et al. (2021) believed that the frother molecules at the air–liquid interface form hydrogen bonds with water to stabilize the liquid film on the surface of the bubbles, thereby preventing the bubbles from coalescing and reducing their size [32].

### 3.2. Effect of Nanobubbles on MFGO Flotation Kinetics

**Figure 4:**
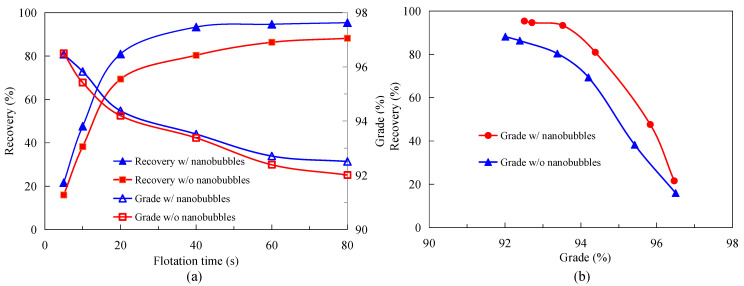
The results of kinetic flotation test with MFPO: (**a**) concentrate recovery and grade as a function of flotation time in the presence and absence of nanobubbles; (**b**) concentrate recovery as a function of concentrate grade in presence and absence of nanobubbles.

Figure 4 shows the results of the kinetic flotation tests performed with and without nanobubbles. It can be seen that the graphite concentrate recovery was significantly higher in the presence of nanobubbles than in their absence at a given flotation time. Moreover, the flotation rate was considerably faster with nanobubbles present in the flotation system, particularly for the first 20 s. The values of the nanobubble and conventional flotation rate constant k calculated based on the flotation data for the first 20 s were 0.025 s^−1^ (i.e., 1.50 min^−1^) and 0.018 s^−1^ (i.e., 1.08 min^−1^), respectively, which indicates that the flotation rate significantly increased with the presence of nanobubbles. The nanobubble flotation process also resulted in a graphite recovery rate that was 7 percentage points higher at the end of the 80 s flotation process than that of the conventional flotation concentrate. Figure 4a shows that the nanobubble flotation process was essentially completed in 40 s, while the conventional flotation process lasted 60 s, indicating that the use of nanobubbles reduced the flotation time by one-third. It is also worth noting from Figure 4a that the nanobubble flotation system consistently produced a higher concentrate grade in addition to a higher rate of recovery than the conventional flotation, suggesting that the nanobubble flotation system was more selective and more effective. The technical advantage of the nanobubble flotation system is better demonstrated by the flotation efficiency curve shown in Figure 4b, which shows that the nanobubble flotation technique always generated a higher rate of flotation recovery than the conventional flotation technique at a given concentrate grade. Previous studies have shown that there are a number of mechanisms by which nanobubbles enhance the flotation separation efficiency with fine particles, as summarized recently by Tao [20]. For example, nanobubbles can promote the agglomeration of fine particles to form agglomerates with a larger apparent size, thus improving the flotation probability of fine particles [39]. In addition, nanobubbles can form preferentially on the surface of the hydrophobic particles, which can increase the difference in surface hydrophobicity between the hydrophobic and hydrophilic particles. The flotation separation process is fundamentally based on [27]. This study reveals a new mechanism by which nanobubbles enhance flotation efficiency, as will be described later.

### 3.3. Effects of Nanobubbles on Diesel Adsorption Kinetics on Graphite

**Figure 5:**
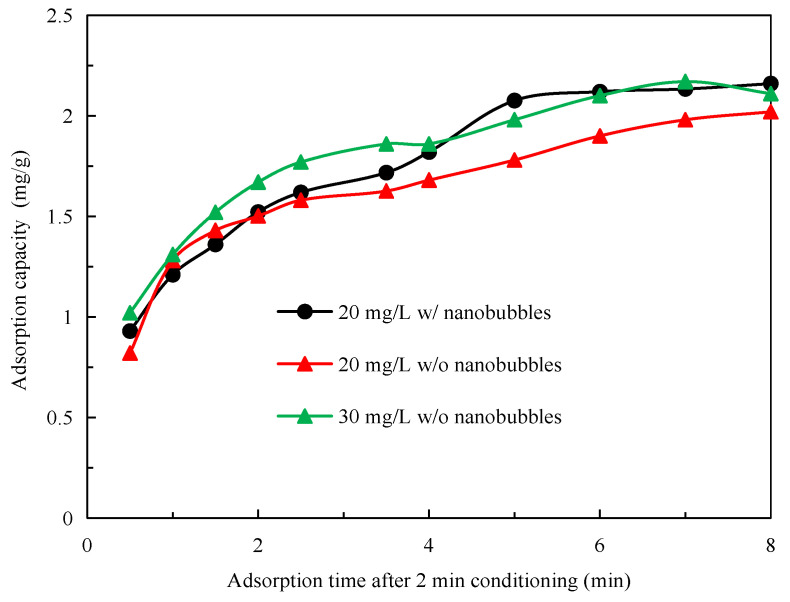
Adsorption capacity of diesel on graphite surface as a function of time after conditioning.

Figure 5 shows the kinetic adsorption results on the graphite surface of the diesel collector at a concentration of 20 and 30 mg/L at pH 10 with and without nanobubbles in the solution, where time 0 represents the moment at which reagent conditioning stopped. The adsorption capacity was not zero at time 0 because adsorption occurred during the reagent conditioning stage. Figure 5 does not show the adsorption capacity change with time during the reagent conditioning stage, which was identical for the nanobubble and conventional flotation techniques. The adsorption capacity of diesel on the graphite surface at a concentration of 20 mg/L was significantly higher at a given adsorption time in the presence of nanobubbles than in their absence. It reached a maximum of 2.08 mg/g in approximately 5 min in the presence of nanobubbles. In contrast, the adsorption capacity reached the maximum of 1.98 mg/g in more than 7 min in the absence of nanobubbles. In other words, the presence of nanobubbles not only improved the diesel adsorption kinetics but also increased its adsorption capacity, which is conducive to improving the mineralization efficiency of graphite particles during flotation.

To better illustrate the effect of nanobubbles on diesel adsorption, Figure 5 also shows the kinetic adsorption behavior of diesel at a concentration of 30 g/L in the absence of nanobubbles. The comparison of the adsorption curve for 20 mg/L diesel in the presence of nanobubbles with the curve for 30 mg/L diesel in the absence of nanobubbles reveals that, although the initial adsorption capacity of diesel was higher at 30 mg/L in the absence of nanobubbles as a result of reagent conditioning, the adsorption capacity of diesel increased more sharply with time in the presence of nanobubbles and became greater after 2 min adsorption time. It reached the maximum adsorption capacity of 2.12 mg/g after 3 min, which was essentially identical to the maximum adsorption capacity achieved after 4 min with 30 mg/L diesel in the absence of nanobubbles. This result is very consistent with the previous studies that have shown that the application of nanobubbles to flotation can considerably reduce the required flotation time and collector dosage by one-third to one-half [10,11,12,13,14,15,16,17,18,19,20,21,22,23,24,25,26,27,28,29,30,31,32,33,34,35,36,37,38,39,40,41,42,43].

**Figure 6:**
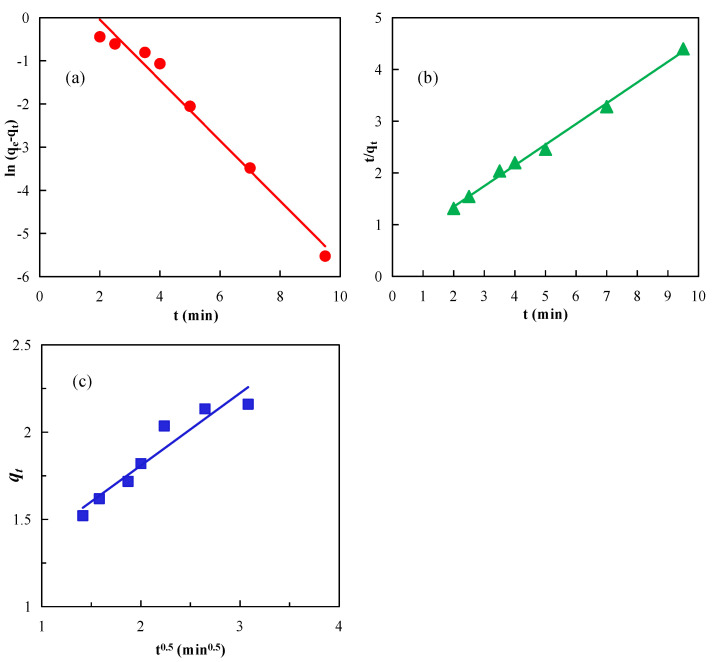
Kinetic adsorption data best fitting with (**a**) quasi-first-order, (**b**) quasi-second-order, and (**c**) intra-particle diffusion models.

The kinetic adsorption behavior of 20 mg/L diesel on the graphite surface in the presence of nanobubbles can be better understood by determining the adsorption isotherm from the data shown in Figure 5 with the quasi-first-order, quasi-second-order, and intra-particle diffusion adsorption models shown, respectively, in Equations (5)–(7) [43,44,45], and the best-fitting results of these models are shown in Figure 6.

(5) $$\ln(q_{e}-q_{t})=\ln q_{e}-k_{1}t,$$

(6) $$\frac{t}{q_{t}}=\frac{1}{k_{2}q_{e}{{}^{2}}_{}}+\frac{t}{q_{e}},$$

(7) $$q_{t}=kt^{0.5}+C,$$

where *t* is the reaction time (min), *q_e_* is the equilibrium adsorption capacity (mg/g), *q_t_* is the adsorption capacity at time *t* moment (mg/g), *k_1_* is the quasi-first-order adsorption rate constant (min^−1^), *k*_2_ is the quasi-second-order adsorption rate constant (g·mg^−1^·min^−1^), *k* is the intra-particle diffusion constant (mg·g^−1^·min^−0.5^), and *C* is the boundary layer thickness (mg/g).

The calculated values of the different adsorption model parameters obtained from Figure 6 are summarized in Table 2. In particular, the values of R^2^, a measure of goodness of fit, were 0.9768, 0.9962, and 0.9151 for the quasi-first-order, the quasi-second-order, and the intra-particle diffusion models, respectively, revealing that the quasi-second-order kinetic model was the best for describing the diesel adsorption process on the graphite surface in the presence of nanobubbles. Stockelhuber et al. (2004) reported that nanobubbles promote hydration film rupture on a hydrophobic surface [46]. The enhanced adsorption of diesel on the graphite surface in the presence of nanobubbles can be attributed to the increased rupture and diffusion of the hydration liquid film caused by the surface nanobubbles on graphite.

### 3.4. Measurement of Surface Potential of Graphite, Diesel Droplets, and Nanobubbles

**Figure 7:**
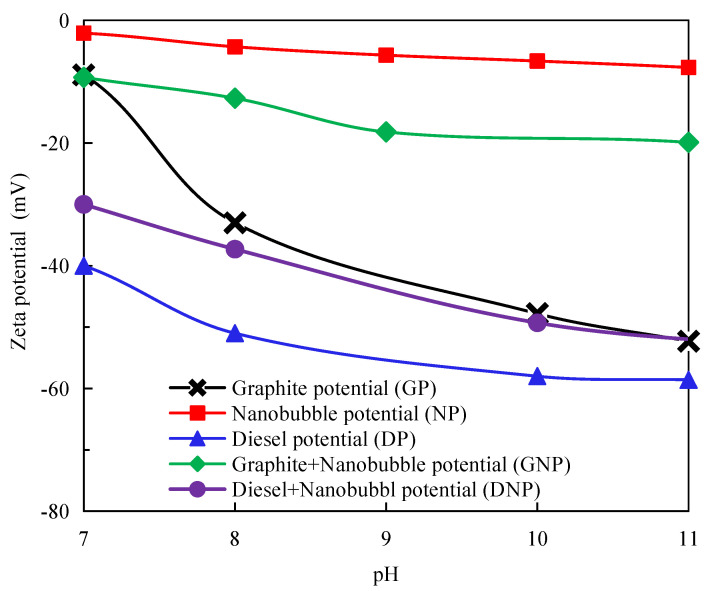
Surface potential of PMFG, nanobubbles, graphite + nanobubbles, diesel, and diesel + nanobubbles.

The surface potentials of graphite, nanobubbles, graphite with nanobubbles, diesel, and diesel with nanobubbles were experimentally measured in this study, and the results are shown in Figure 7. The value of the negative surface potential of all the samples significantly increased with increasing pH. The surface of graphite does not absorb any ions or dissolve inevitable ions since graphite is a non-polar mineral. The isoelectric point of graphite should be around pH 7 if the crystal of graphite is intact. It is believed that lattice defects on a graphite surface formed during graphite’s mineralization and grinding process are primarily responsible for the deviation of the isoelectric point of the graphite surface [47]. Moreover, lattice defects can result in oxygen-containing groups attached to the graphite surface in aqueous solutions, which hydrolyze to create the graphite surface charge. Oxygen-containing groups such as C-O and C=O are naturally hydrophilic and reduce surface hydrophobicity, resulting in adverse effects on flotation. Conversely, a graphite surface with a complete lattice is dominated by the C-C functional groups, which belong to hydrophobic functional groups [48].

**Figure 8:**
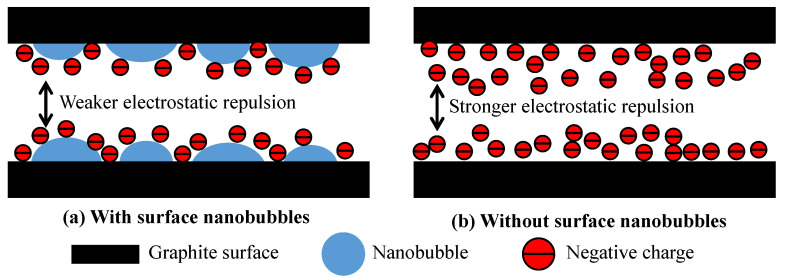
Illustration of surface nanobubbles reducing electrostatic repulsion between graphite.

The nanobubble surface-charging mechanisms are known to be related to the molecular structure of the frother or surfactant added to the solution [49]. When a non-ionic surfactant such as fusel is present in the solution, the surface charge of nanobubbles is pH-dependent. Figure 7 shows that the nanobubbles possessed a relatively low negative potential under alkaline conditions, whereas the graphite particles showed a substantially greater surface potential in the absence of nanobubbles. A pronounced reduction in the surface potential of the graphite particles and diesel occurred in the presence of nanobubbles, as shown in Figure 7. This reduced the electrostatic repulsion between the graphite particles and promoted their agglomeration, improving the flotation efficiency. The difference in the electrostatic repulsion between the graphite particles with (Figure 8a) and without the surface nanobubbles (Figure 8b) is illustrated in Figure 8. The substantial decrease in the graphite surface potential in the solution with nanobubbles is because the surface nanobubbles that are formed during hydrodynamic cavitation mask the oxygen-containing groups on the graphite surface.

**Figure 9:**
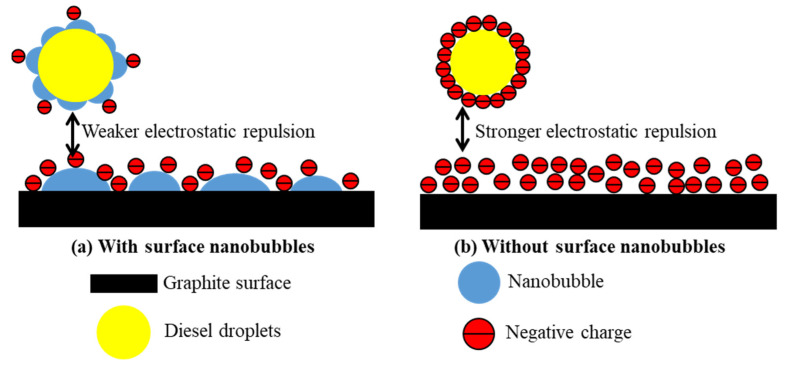
Illustration of electrostatic repulsion between diesel droplets and graphite surface with (**a**) and without (**b**) nanobubbles.

In the system containing fusel, diesel is more likely to be emulsified to form tiny oil droplets via hydraulic cavitation. He et al. obtained 150–400 nm oil droplets via hydraulic cavitation in a mixed system of alcohol and diesel [50]. Figure 7 shows that the emulsified diesel droplets possessed very high negative surface potential in the absence of nanobubbles that increased with the solution’s pH, but the surface potential of diesel significantly decreased with the presence of nanobubbles. Similar to graphite particles, diesel droplets are non-polar and hydrophobic, and nanobubbles can easily form on the surface of diesel droplets to significantly reduce their surface potential. Thus, the electrostatic repulsion between the diesel surface and the graphite surface can be substantially reduced in the presence of nanobubbles, as illustrated in Figure 9a, increasing the adsorption of diesel on the graphite surface and improving the hydrophobicity of the graphite surface. In contrast, graphite and diesel surfaces are characterized by a very high negative potential in the absence of nanobubbles, resulting in a strong electrostatic repulsion, as illustrated in Figure 9b, which is not conducive to the adsorption of diesel on the graphite surface.

### 3.5. FTIR Characterization of Diesel Adsorption on Graphite Surface

**Figure 10:**
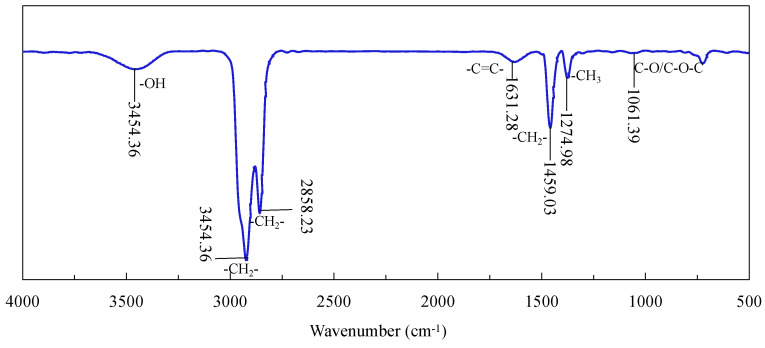
Infrared spectrum measurement results of diesel.

Figure 10 shows the FTIR spectra of diesel. It can be seen that the stretching vibration characteristic peak of -OH appeared at 3455.94 cm^−1^; the antisymmetric stretching vibration characteristic peak of -CH_2_- appeared at 2923.43 cm^−1^; the symmetric stretching vibration characteristic peak of -CH_2_- appeared at 2857.88 cm^−1^; the stretching vibration characteristic peak of -C=C- appeared at 1631.28 cm^−1^. The peaks at 1458.55 cm^−1^ and 1375.34 cm^−1^ may be characteristics of the asymmetric bending vibration of -CH_2_- and the symmetric bending vibration of -CH_3_, respectively, whereas the peak at 1061.39 cm^−1^ may be a characteristic of the stretching vibration of C-O or C-O-C from the trace impurities in diesel.

**Figure 11:**
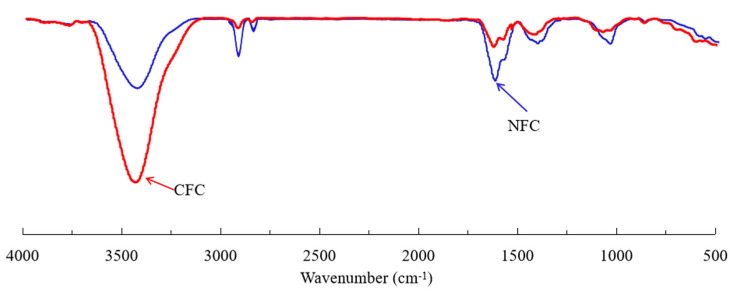
FTIR measurement results of NFC and CFC.

It has been suggested that the hydrophobic degree of a mineral surface can be evaluated through the intensity of the -OH peak on infrared spectra, with a weaker -OH peak representing a more hydrophobic surface [51]. Figure 11 shows the infrared spectra of CFC and NFC. It can be seen that the -OH peak near 3500 cm^−1^ for the NFC surface was significantly weaker than that for CFC, while the above-discussed characteristic diesel peaks, especially those near 1418 cm^−1^, 1630 cm^−1^, and 1050 cm^−1^, were stronger with NFC than with CFC, indicating that the introduction of nanobubbles enhanced the adsorption of diesel on the graphite surface and achieved a higher degree of hydrophobicity, which is consistent with the kinetic adsorption data shown in Section 3.3.

**Figure 12:**
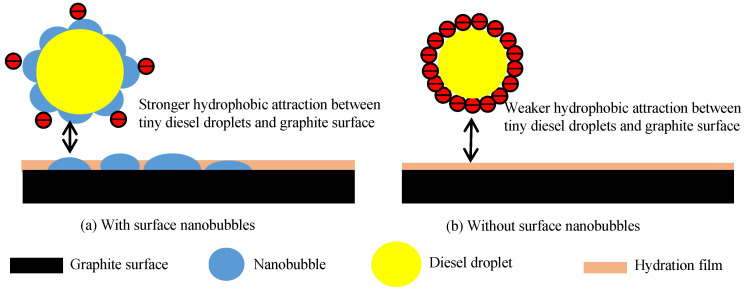
Illustration of hydrophobic attraction between graphite and diesel droplets with (**a**) and without nanobubbles (**b**).

The enhanced hydrophobicity of graphite surfaces by nanobubbles is related to the enhanced hydrophobic interaction between the diesel droplets and mineral surfaces. Stockelhuber et al. (2004) reported that the nanobubbles preferentially formed on mineral surfaces could promote hydration film rupture on the mineral interface [43]. Calgaroto et al. (2015) demonstrated that the surface nanobubbles significantly increased the mineral surface hydrophobicity [52]. It should also be mentioned that hydrodynamic cavitation can promote the existence of diesel in the slurry as tiny droplets [50], which can be considered oily bubbles since the surface properties of oil droplets and oily bubbles are essentially identical [36]. The extended DLVO theory reveals that hydrophobic attraction is the major driving force behind the mineralization of oily bubbles through the adhesion of hydrophobic mineral surfaces [36], and a more hydrophobic mineral surface results in a stronger hydrophobic attraction between the oil droplets and the mineral surface. Figure 12 illustrates how nanobubbles promote hydration film rupture and enhance the hydrophobic attraction between the diesel droplets and the graphite surface. Nanobubbles rupture and displace the liquid films on the graphite surface and, consequently, enhance the hydrophobic attraction between diesel and graphite (Figure 12a). This hydrophobic attraction is far greater than the electrostatic repulsion between diesel and graphite (Figure 12b), leading to the strong adsorption of diesel on the graphite surface. Less liquid film and more diesel adsorbed on the graphite surface are responsible for the weaker -OH peak intensity on NPC than on CFC. In contrast, in the absence of nanobubbles, there is a weaker hydrophobic attraction between diesel droplets and graphite surfaces, resulting in the poor adsorption of diesel on graphite surfaces and, consequently, the lower hydrophobicity of the mineral.

It should be noted that the enhancement in the hydrophobic attraction between diesel and graphite due to the presence of nanobubbles can significantly improve the hydrophobicity of the graphite surface, which facilitates hydrophobic agglomeration and improves the stability of the hydrophobic aggregates of fine graphite particles. The stable graphite aggregates existing in the pulp with larger apparent sizes significantly improve the recovery of fine graphite particles as a result of the improved probability of collection during flotation [31]. It should also be mentioned that there is a capillary bridge relay between the two surface nanobubbles, and this capillary mechanism can promote the formation of a “bridge” between the nanobubbles on the diesel surface and the nanobubbles on the graphite surface, thus promoting the adsorption of diesel oil on the surface of graphite. However, the nature of this capillary force is currently unknown. Based on the results of this study, the hydrophobic force and electrostatic force may be important factors in promoting this capillary mechanism.

### 3.6. Contact Angle Measurements

**Figure 13:**
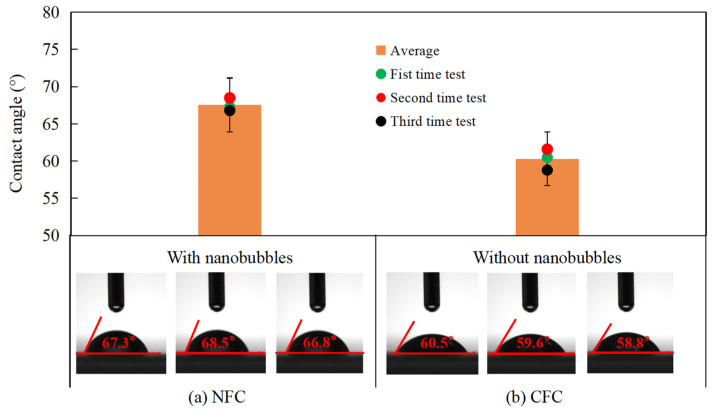
Contact angle measurements of NFC (**a**) and CFC (**b**).

The contact angle measurements of CFC and NFC were carried out to quantify the effect of nanobubbles on the hydrophobicity of the graphite surface, and the results are shown in Figure 13. It can be seen that the average value of the NFC surface contact angle (Figure 13a) was approximately 8° larger than that of the CFC surface (Figure 13b), i.e., 68° vs. 60°. The application of nanobubbles in flotation increased the hydrophobicity of the graphite surface, which is consistent with the above characterization results and fundamental analyses. It should be noted that the contact angle was measured ex situ after the concentrate particles from flotation were washed and filtered. In other words, the nanobubbles were not present on the NFC particles used for contact angle measurements even though the NFC particles were nanobubble flotation products, suggesting that the observed increase in the contact angle is solely a result of the increased adsorption of diesel on the particles.

**Figure 14:**
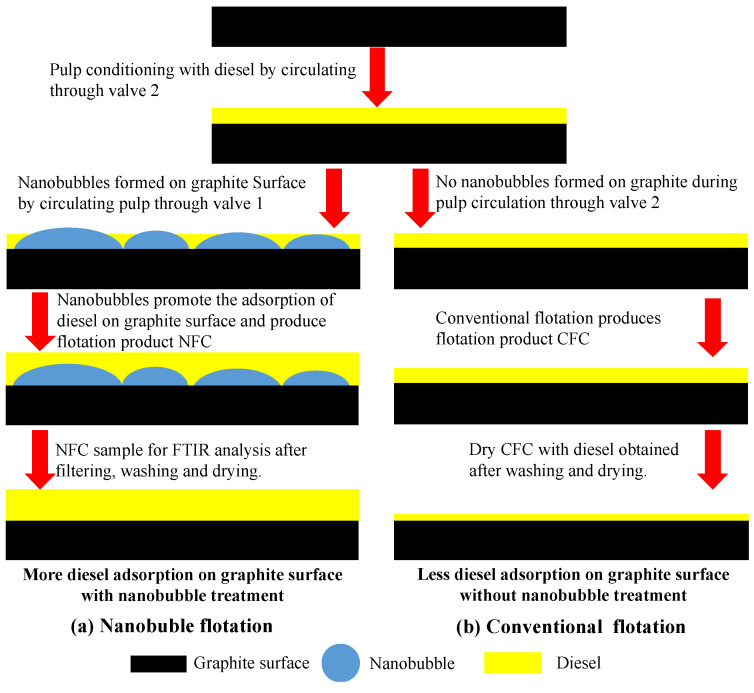
Illustration of effects of surface nanobubbles on entire flotation process of graphite.

In summary, the differences between the nanobubble flotation (Figure 14a) and conventional flotation process (Figure 14b) can be described in Figure 14. During the reagent conditioning stage, before the nanobubbles were generated, diesel adsorption occurred on the graphite surface, providing hydrophobic sites for the initial formation and subsequent growth of the nanobubbles when the hydrodynamic cavitation process was initiated. The formation of the surface nanobubbles further increased the adsorption of diesel on the graphite surface as a result of the reduced electrostatic repulsion and increased hydrophobic attraction. An increased amount of diesel was still present on the NFC particles collected after flotation followed by washing and drying.

## 4. Conclusions

Based on the above description and discussion of our experimental results on the comparative flotation kinetics, adsorption capacity, Zeta potential, FTIR, and contact angle with graphite particles under various conditions with and without nanobubbles, the following conclusions can be drawn from this study:

(1) The graphite flotation results showed that the flotation kinetics and the rate of recovery and the grade of the concentrate were significantly enhanced by the presence of nanobubbles;
(2) The presence of the surface nanobubbles increased the adsorption rate and capacity of diesel on the graphite surface, significantly improving its hydrophobicity. The mineralization efficiency of the flotation process was also significantly improved by the surface nanobubbles, which is partly responsible for the increased graphite flotation kinetics and selectivity;
(3) The nanobubbles formed on the surface of the graphite compound effectively reduced the electrostatic repulsion between the graphite particles, promoting the agglomeration of fine graphite particles and increasing the stability of the graphite agglomerates. The surface nanobubbles also reduced the electrostatic repulsion between the diesel droplets and graphite particles and increased the adsorption capacity of diesel on the graphite surface, which improved the degree of hydrophobicity of the graphite surface and the selectivity of flotation;
(4) The FTIR results and contact angle measurements confirmed that the surface nanobubbles improved the hydrophobicity of the graphite surface, increased the hydrophobic attraction between the graphite particles and diesel droplets and the adsorption capacity of diesel on the graphite surface, further improving the degree of the hydrophobicity of the graphite surface;
(5) Future studies are needed to investigate how nanobubbles function to mask the hydrophilic sites on graphite surfaces. The interactions of nanobubbles with oil droplets and the consequent effects on oil adsorption on graphite should also be studied to achieve a better understanding of the fundamentals of nanobubble-enhanced flotation.

## Figures and Tables

**Table 1:** Mineral compositions of MFGO.

Mineral	Graphite	Quartz	Muscovite	Pyrite	Garnet	Calcite	K-Feldspar
Content (%)	84.06	3.47	4.78	2.69	1.12	1.07	0.98

**Table 2:** Calculation results of adsorption kinetic equation parameters.

Model	Linear Fitting Equation	Parameter	Parameter Values
Quasi-first-order dynamics	y = −0.6996x + 1.3511	*q_e_*	2.1636
		*q*_*e*1_	3.8617
		*k*_1_	0.6996
		*R* ^2^	0.9768
Quasi-second-order dynamics	y = 0.4004x + 0.5452	*q_e_*	2.1636
		*q*_*e*1_	2.4975
		*k*_2_	0.2941
		*R* ^2^	0.9962
Intra-particle diffusion	y = 0.4141x + 0.9808	*k*	0.4141
		*C*	0.9808
		*R* ^2^	0.9151

*Note:* *q_e_ and* *q_e1_ are the experimental and calculated equilibrium adsorption capacities, respectively.*

## Data Availability

Data available on request due to restrictions.
